# Supplementary material for: Similarity of stream width distributions across headwater systems
Source: Nat Commun. 2018 Feb 9;9:610. doi: 10.1038/s41467-018-02991-w (PMC5807321; doi:10.1038/s41467-018-02991-w)
Supplement: Supplementary file 1 — Supplementary Information [file 41467_2018_2991_MOESM1_ESM.pdf]

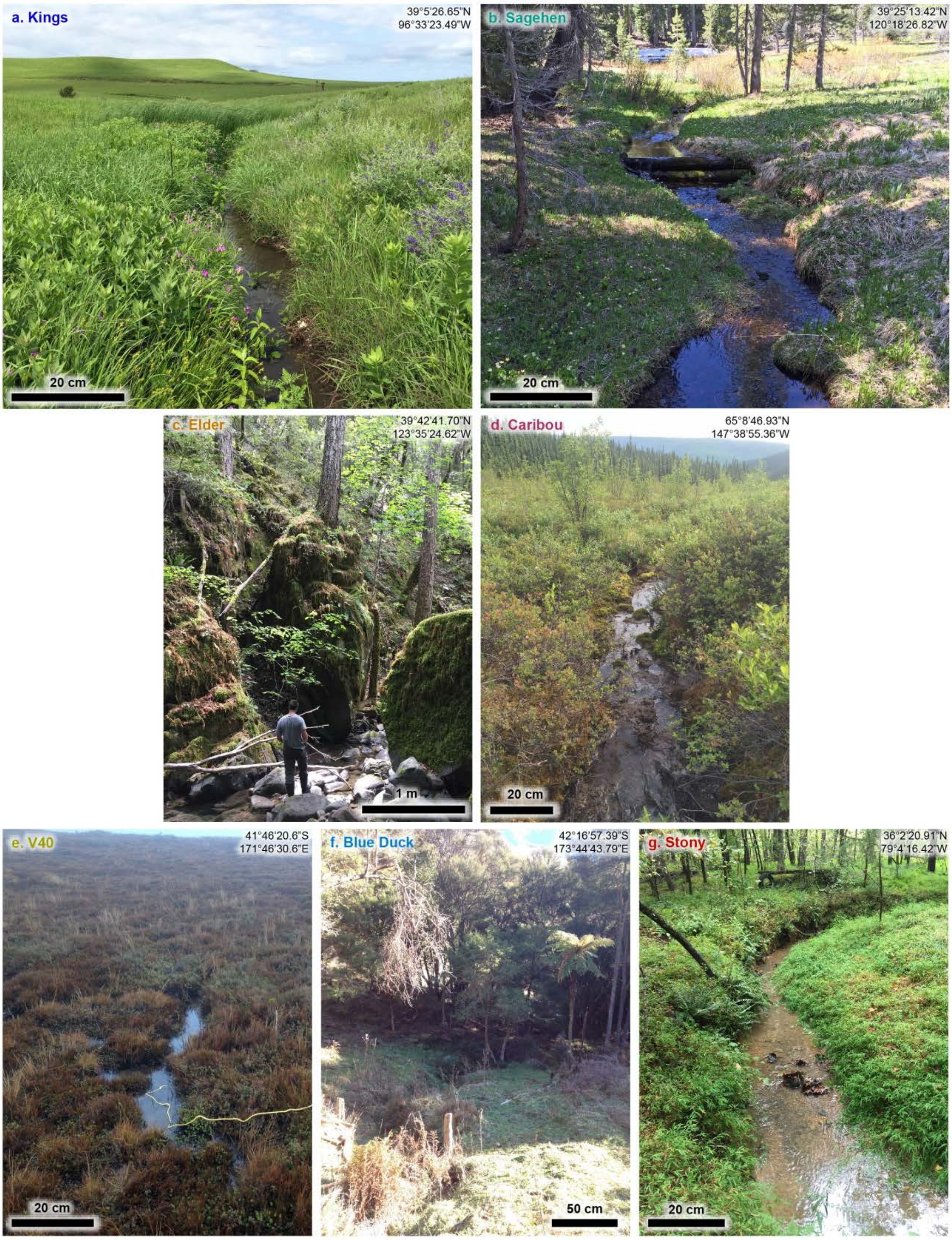

Supplementary Figure 1 | Photographs of study catchments and streams. a-h, We surveyed supply- and transport-limited bedrock streams, streams with alluvial substrates (clay, sand, glacial till), and streams impacted by vegetation and woody debris. Study sites also included perennial spring-fed streams, intermittent or ephemeral streams, and streams flowing over permafrost.

Catchment	North Branch Kings Creek (K1B tributary)	Upper Sagehen Creek sub-catchment	Upper Elder Creek	C1 tributary of Caribou Creek	V40 Stream sub-catchment	Blue Duck Creek sub-catchment	Stony Creek Research Watershed
**Location**	Konza Prairie Biological Station, KS	Sagehen Creek Field Station, CA	Angelo Coast Range Reserve, CA	Caribou-Poker Crk. Research Watershed, AK	V40 Stream, NZ	Blue Duck Creek, NZ	Duke Forest, NC
**Date Surveyed**	May 25-26, 2015	May 29-30, 2015	Jun 1-2, 2015	Jun 20, 2015	Jun 28-29, 2015	Jul 12-14, 2015	Sep 27, 2015
**Surveyors**	G Allen, A Tashie	G Allen, A Tashie	G Allen, A Tashie	G Allen, D Butman	E Barefoot	E Barefoot, E Beckham	G Allen, E Barefoot
**Outlet Lat. (DD)**	39.0972	39.433	39.718	65.1486	-41.77	-42.278	36.04
**Outlet Lon. (DD)**	-96.5714	-120.285	-123.604	-147.637	171.777	173.746	-79.067
**Drainage Area (ha)**	180	449	362	258	6	56	128
**Altitude Range (m)**	371-443	2,083-2,661	574-1,250	401-657	699-741	136-400	158-226
**Stream Network Relief (m)**	21.5	131.6	160.2	89	39.5	95.2	36.9
**N Width Obs.**	1,797	1,422	1,044	363	249	519	805
**ADN Length (km)**	8.99	7.11	5.22	1.82	1.25	2.6	4.03
**ADN Drainage Density (km^{-1})**	4.99	1.58	1.44	0.7	20.75	4.63	3.15
**% Basin Stream Surface Area**	0.46	0.12	0.14	0.02	0.91	0.4	0.31
**1st Order Median Width (cm)**	30.5	22.6	35.6	20.3	22.9	23.4	30.5
**Mode Width (cm)**	34.6	25.2	27.4	27	21.5	21.6	32.7
**Gage**	USGS gage	USGS gage	USGS gage	CPCRW gage	West Coast Regional Council gage	Environment Canterbury gage	Duke Forest Research Watershed
**Gage Location**	Kings Creek near Manhattan (downstream from catchment)	Sagehen Creek near Truckee (downstream from catchment)	Elder Creek near Branscomb (downstream from catchment)	Caribou Creek near Poker Flat (downstream from catchment)	Buller River at Te Kuha (on a nearby river)	Lyell Creek at Warren Creek Confluence (on a nearby creek)	Stony Creek near Hillsborough (at bottom of study catchment)
**Gage Drainage Area (km^2)**	10.6	27.2	16.8	104	6,350	64	1.3
**Gage Discharge (m^3/s)**	0.30±0.04	0.062±0	0.057±0	0.137±0	419±8.7	0.156±0	0.019±0
**Flow Record Length (yrs)**	36	62	48	11	6	12	3
**Flow Percentile (1σ)**	87.4±8.4	19.4±1.8	32.1±0.9	6.6±0	73.1±0.6	2.3±0.5	53.1±0
**DEM Source**	FEMA 2006 LIDAR	NED	NED	GDEM V2	LINZ	LINZ	NCFMP LIDAR
**DEM Resolution (m)**	2	10	10	15	8	8	6
**Bedload Grain Size Range**	Sand-cobble	Gravel-cobble	Gravel-boulder	Gravel-cobble	Sand-cobble	Gravel-cobble	Gravel-cobble
**Lithology**	Interbedded mudstone and limestone	Siliceous intrusives and metamorphics with glacial deposits	Sandstone and mudstone	Greenschist and loess deposits	Interbedded coal and sandstones	Sandstones and mudstone	Intrusives and meta-sediments
**Climate**	Continental climate with wet summers, cold winters	Continental subarctic with cold winters	Medi-terranean	Continental subarctic	Temperate maritime	Temperate dry	Humid subtropical
**Vegetation**	Native tallgrass prairie with deciduous forest in valley bottom	White alder bushes, wet montane meadows and conifer forests	Old-growth Douglas-fir forest	Black spruce/feather moss slopes and treeless muskeg in valley bottom	Disturbed temperate rainforest. Native beech and tussock grass.	Mixed pasture grasses and native mixed beech and fern forest	Formerly farmed oak and hickory forest
**Notes**	Annual controlled burn	Formerly glaciated, spring fed	Spring fed, high rock uplift rates	Permafrost on north-facing slopes	Former coal mine	Cattle grazed	Partially developed, natural piping

Supplementary Table 1 | Attributes of the seven physiographically contrasting study catchments. Note: the Caribou Creek gage station is operational during summer months only. FEMA 2006 LIDAR DEM source: USGS, Federal Emergency Management Agency 2006 LIDAR (2006), see: http://www.kansasgis.org/catalog/_cat_metadata.cfm?meta_id=223. NED source: USGS, National Elevation Dataset (2009), see: https://lta.cr.usgs.gov/NED. GDEM V2 source: ASTER Global Digital Elevation Map (2011), see: https://asterweb.jpl.nasa.gov/gdem.asp. LINZ source: Land Information New Zealand New Zealand 8m Digital Elevation Model (2012), see: https://data.linz.govt.nz/. NCFMP LIDAR source: North Carolina Floodplain Mapping Program 2007 LIDAR (2007), see: http://www.nconemap.com.

Date Surveyed	2015/10/27	2015/12/09	2016/02/02	2016/02/14	2016/03/04a	2016/03/04b
Surveyors	E Barefoot,	E Barefoot,<br>E Henry	E Barefoot	E Barefoot	E Barefoot,<br>A Tashie	E Barefoot,<br>A Tashie
N Width Obs.	160	368	514	428	531	535
Mode Width (cm)	35.5	35.0	41.7	36.1	39.0	38.5
ADN Length (m)	800	1840	2570	2140	2655	2675
ADN Drainage Density (km^{-1})	1.65	3.80	5.31	4.42	5.49	5.53
1st Order Median Width (cm)	33.0	33.0	43.2	39.4	40.6	40.6
% Basin Stream Surface Area	0.11	0.25	0.42	0.28	0.42	0.43
Discharge (L/s)	7.5	14.5	15.3	8.8	13.9	14.3
Catchment-averaged Runoff (mm/day)	1.5	2.6	2.9	1.7	2.5	2.5
Flow Percentile (%)	53	73	78	57	72	72

Supplementary Table 2 | Attributes of the six repeat stream width surveys in the Stony subcatchment. Stream gage is located at the outlet of the surveyed subcatchment. The Stony subcatchment has a drainage area of 48 ha and an elevation ranging from 163 to 210 m.

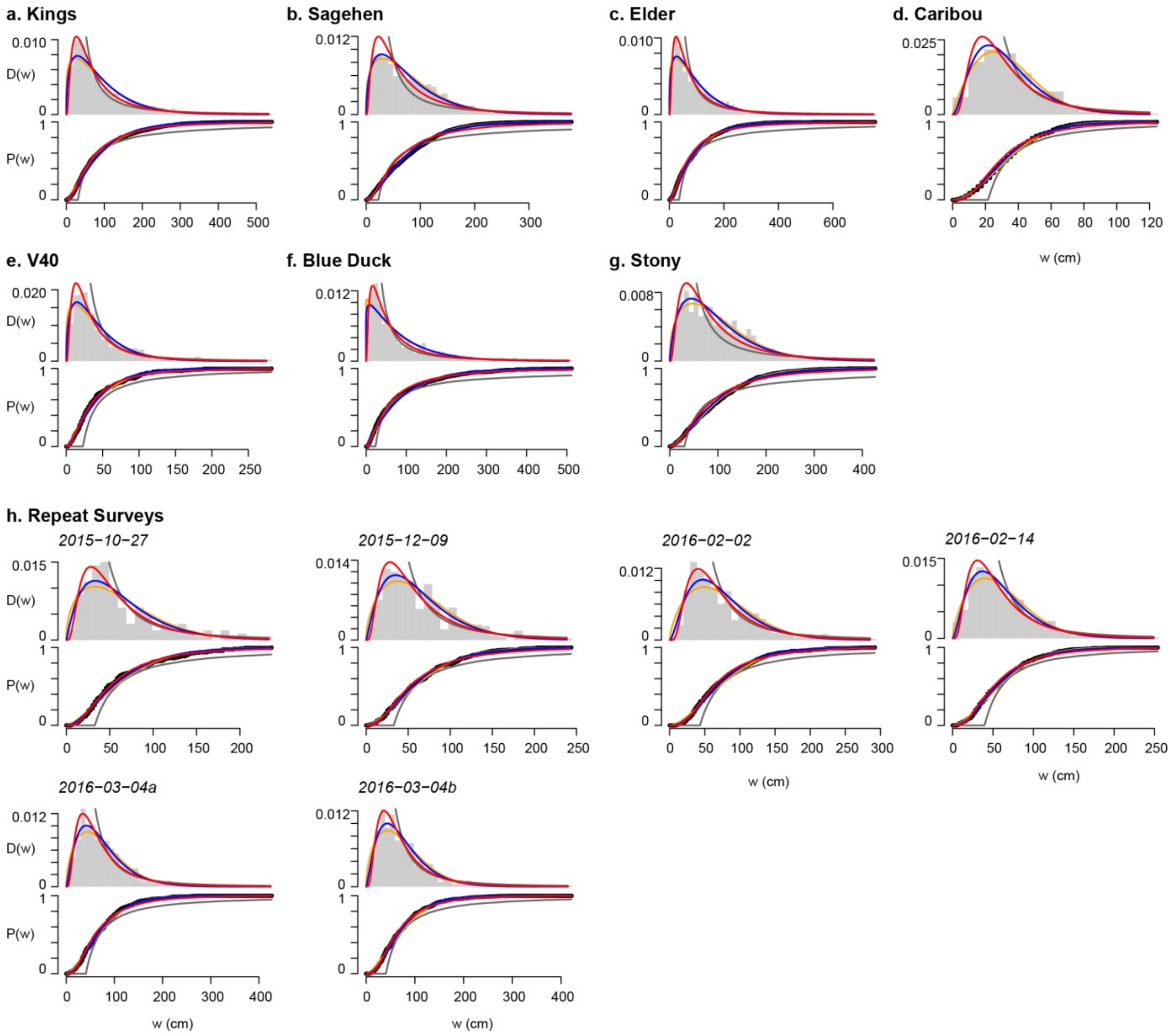

Supplementary Figure 2 | Fitted distributions to stream width data. a-h, The upper panels show histograms with probability density functions and the lower panels show cumulative distribution functions. Unimodal distributions (lognormal, gamma and Weibull) describe stream width data better than the Pareto distribution in all study catchments. See Supplementary Tab. 3 for statistics of fits. To improve goodness of fit, Pareto distributions were fit to data greater than the median first-order stream width after Allen and Pavelsky[21].

Statistical Parameter	Kings	Sagehen	Elder	Caribou	V40	Blue Duck	Stony	2015/ 10/27	2015/ 12/09	2016/ 02/02	2016/ 02/14	2016/ 03/04a	2016/ 03/04b
**Lognormal Distribution**													
**location, μ**	4.153	4.01	4.17	3.363	3.417	3.939	4.3	3.912	3.938	4.149	3.916	4.081	4.108
**scale, σ**	0.927	0.944	0.983	0.659	0.892	1.068	0.867	0.749	0.758	0.656	0.688	0.739	0.697
**X^2**	81.6	166.3	95.7	37.4	28.1	57.3	103.1	22.3	52	42.8	92.8	70.9	44.2
**X^2 p**	0.018	0.001	0.099	0.001	0.418	0.282	0.001	0.496	0.001	0.086	0.001	0.023	0.333
**K-S D**	0.028	0.081	0.061	0.08	0.047	0.039	0.083	0.041	0.063	0.044	0.061	0.061	0.05
**K-S p**	0.129	<0.001	0.001	0.019	0.628	0.397	<0.001	0.948	0.107	0.266	0.079	0.037	0.139
**Gamma Distribution**													
**shape, k**	1.463	1.573	1.382	2.888	1.512	1.109	1.809	2.074	2.186	2.639	2.573	2.264	2.378
**rate, β**	0.016	0.02	0.014	0.083	0.034	0.013	0.018	0.032	0.033	0.034	0.042	0.03	0.031
**X^2**	131	45.8	211.5	12.2	91.2	101.5	45.5	28.4	40.1	53.3	19.1	129.4	152.1
**X^2 p**	0.001	0.157	0.008	0.434	0.009	0.005	0.313	0.185	0.041	0.01	0.741	0.01	0.007
**K-S D**	0.062	0.041	0.041	0.043	0.077	0.071	0.035	0.078	0.035	0.05	0.034	0.038	0.046
**K-S p**	<0.001	0.017	0.065	0.513	0.101	0.011	0.29	0.278	0.764	0.148	0.703	0.435	0.202
**Weibull Distribution**													
**shape, k**	1.215	1.336	1.192	1.866	1.215	1.026	1.44	1.47	1.569	1.671	1.704	1.562	1.571
**scale, λ**	99.5	85.2	103.1	39.1	47.1	86.9	109.9	72.1	73.3	87.2	69.4	83.8	85.3
**X^2**	164.6	37.8	376.4	19.6	113.6	96.3	51.6	35	50.1	88.3	51.3	1287.2	1113.1
**X^2 p**	0.001	0.408	0.002	0.105	0.006	0.005	0.163	0.066	0.009	0.002	0.037	0.002	0.001
**K-S D**	0.064	0.034	0.038	0.038	0.083	0.06	0.031	0.089	0.053	0.061	0.049	0.041	0.052
**K-S p**	0	0.069	0.101	0.666	0.064	0.045	0.422	0.158	0.262	0.042	0.26	0.323	0.11
**Pareto Distribution**													
**scale, x_m**	30.48	22.60	35.56	21.59	22.86	23.368	30.48	33.02	33.02	43.18	39.37	40.64	40.64
**shape, α**	0.914	0.823	0.923	1.623	1.211	0.792	0.852	1.256	1.242	1.385	1.596	1.309	1.307
**X^2**	316	712	237.3	87.6	45.9	135.5	346.1	35.5	124.5	110.1	57.4	109.7	106.6
**X^2 p**	0.001	0.001	0.001	0.001	0.029	0.001	0.001	0.031	0.001	0.001	0.001	0.001	0.001
**K-S D**	0.156	0.209	0.183	0.19	0.106	0.162	0.212	0.151	0.173	0.162	0.176	0.184	0.171
**K-S p**	<0.001	<0.001	<0.001	<0.001	<0.001	<0.001	<0.001	<0.001	<0.001	<0.001	<0.001	<0.001	<0.001
**Width Model (Fig. 3)**													
**location, μ**	4.268	4.402	4.689		3.944	4.313							
**scale, σ**	0.755	1.077	0.686		0.733	0.622							
**K-S D**	0.081	0.324	0.253		0.164	0.134							
**K-S p**	<0.001	<0.001	<0.001		<0.001	<0.001							

Supplementary Table 3 | Statistics of distribution fits. Lognormal, gamma, Weibull, and Pareto distribution maximum likelihood estimated statistical parameters with Pearson's X^2 statistic and corresponding *p*-value, and with two sided one sample Kolmogorov-Smirnov (K-S) statistic (*D*) and corresponding *p*-value.

Survey	Kings Creek (K1B Tributary)	Sagehen Creek Subcatch-ment	Upper Elder Creek	Caribou Creek (C1 Tributary)	Stony Creek Research Watershed	Stony Creek Subcatch-ment 27/10/2015	Stony Creek Subcatch-ment 09/12/2015	Stony Creek Subcatch-ment 02/02/2016	Stony Creek Subcatch-ment 14/02/2016	Stony Creek Subcatch-ment 04/03/2016a	Stony Creek Subcatch-ment 04/03/2016b
**Observed Stream Length (km)**	8.98	7.11	5.22	1.82	4.03	0.8	1.84	2.57	2.14	2.66	2.68
**Runoff (mm/day)**	2.47	0.14	0.46	0.11	0.50	1.34	2.59	2.73	1.57	2.48	2.55
**Flowline Dataset**	NHDPlus V2	NHDPlus V2	NHDPlus V2	EDNA	NHDPlus V2	NHDPlus V2	NHDPlus V2	NHDPlus V2	NHDPlus V2	NHDPlus V2	NHDPlus V2
**HUC Region**	HUC 10	HUC 18 Dry	HUC 17	AK HUC 4	HUC 3	HUC 3	HUC 3	HUC 3	HUC 3	HUC 3	HUC 3
**Flowline Length (km)**	1.9	2.68	3.19	0.878	0	0	0	0	0	0	0
**Observed Surface Area (SA) (m^2)**	8351	5568	5059	630	4011	519	1207	1991	1321	1992	2037
**Eq. (1) Derived SA (m^2)**	6609	5226	3840	1335	2963	589	1353	1892	1574	1955	1967
**Flowline Derived SA (m^2)**	610	8269	9340	940	0	0	0	0	0	0	0
**CO_2 Flux from Observed SA (Mg-C/Yr)**	26.2 (9.2-45.1)	65.6 (18.3-91.7)	49.6 (13.6-70.4)	2.1 (0.8-3.6)	9.7 (3.3-15.7)	1.3 (0.43-2.1)	3.2 (1.1-5.2)	5.2 (1.7-8.3)	3.33 (1.1-5.2)	5.24 (1.8-8.6)	5.2 (1.8-8.6)
**CO_2 Flux from Eq. (1) SA (Mg-C/Yr)**	21.4 (7.2-35.1)	62 (17.0-87.2)	37.7 (10.6-53.6)	4.6 (1.8-7.9)	7.0 (2.4-11.6)	1.4 (0.5-2.3)	3.6 (1.2-5.7)	4.9 (1.7-8.1)	3.77 (1.3-6.2)	5.17 (1.7-8.2)	5.3 (17-8.4)
**CO_2 Flux from Flowline SA (Mg-C/Yr)**	1.9 (0.7-3.2)	97.2 (26.7-137.2)	90.4 (25.6-131)	3.3 (1.2-5.5)	0	0	0	0	0	0	0

Supplementary Table 4 | Carbon dioxide efflux calculation attributes. Values of CO_2 efflux are calculated using three metrics of stream surface area: 1) the observed surface area measured in the field; 2) the surface area calculated by applying Eq. (1) on the observed stream lengths; and 3) the surface area derived from DEM flowline datasets. Numbers in parentheses represent 5th and 95th percentile ranges from Monte Carlo simulations (see Methods).
